# Peer education is a feasible method of disseminating information related to child nutrition and feeding between new mothers

**DOI:** 10.1186/1471-2458-14-1262

**Published:** 2014-12-12

**Authors:** Kerith Duncanson, Tracy Burrows, Clare Collins

**Affiliations:** Nutrition and Dietetics, School of Health Sciences, Faculty of Health, The University of Newcastle, University Drive, Callaghan, NSW 2308 Australia; Priority Research Center in Physical Activity and Nutrition, The University of Newcastle, Callaghan, NSW 2308 Australia; 117 Becker Road, Forster, NSW 2428 Australia

## Abstract

**Background:**

This study examined whether peer education based on the Theory of Planned Behaviour is a feasible method to share and disseminate nutrition and feeding information between mothers of babies and toddlers.

**Methods:**

The Peer Educator Nutrition Training (PeerENT) study was a feasibility study. Participants were recruited from an existing cohort of mothers of six month to two year olds. An online survey tool was used to collect and collate data, which was then analysed using STATA statistical software.

**Results:**

Thirty four mothers (35%) responded to the survey with 76% (n = 26) either very interested (n = 13) or interested (n = 13) in receiving child nutrition information from a trained peer educator, preferably in a structured group session. Sixty five per cent (n = 22) were “interested” or “very interested” in becoming a peer nutrition educator. The preferred methods of communicating information to other parents were online (n = 17), informally in a social group (n = 16) and via a face-to-face group program (n = 14). Participants predicted they would share child nutrition information with an average of fifteen people, a total reach of 510 individuals.

**Conclusions:**

High levels of interest in peer educator training and the capacity for mothers to share resources widely and easily via social media offers a potential opportunity to disseminate evidence-based nutrition information. A pilot study investigating the impact of a well-designed, theory-based peer nutrition education program on the child feeding practices of mothers with children aged between six months to two years is warranted.

## Background

The nutritional quality and variety of a young child’s dietary intake is heavily influenced by the feeding practices of their parents [1–4], particularly in the first five years of life. Parental factors including life experience, health status, education and self-efficacy impact on child feeding, in combination with interpersonal relationships and environmental factors such as geographic location and food costs [1, 5, 6].

Parents of children under the age of two years are considered to be particularly receptive to knowledge and skill development around parenting and the promotion of healthy family eating and physical activity behaviours [7]. High levels of concern regarding children’s appetite, eating patterns and growth are commonly reported topics addressed by health professionals [8], and parents regularly express the desire for more comprehensive guidance in these areas [9].

While health professionals continue to be highly utilised and influential sources of nutrition information for parents of young children, emerging evidence suggests that parents are more likely to change their child-feeding practices as a result of peer influence, rather than through education by health professionals [10]. Conceptually, this can be explained within the Theory of Planned Behaviour (TPB) [11] framework in which an individual’s behaviour is determined by complex interactions between their beliefs and attitudes. Of particular relevance are the TPB constructs of individual perceptions about relevant others’ beliefs (subjective norms) and influences of significant others on beliefs (normative beliefs). Normative beliefs and resulting subjective norms of parents in relation to child-feeding are largely attributable to the peer environment of the parent [12].

New mothers can be one of the most socially isolated groups in communities [7], potentially increasing mental and physical health risk for these women and their families. Such social isolation may be exacerbated by geographic isolation of rurality [13, 14]. In Australia, this potential social isolation is addressed in communities through the establishment of New Parent Groups [7] by Early Childhood Nurses [15], who have regular contact with the mothers and babies. From the time of birth of a parent’s first child, strong social connections form between parents with infants of a similar age [16]. The parents’ peers become their support network, providing social connectedness, a source of shared information and peer education [15, 16].

Peer health education is the process of sharing health related information from person to person among members of a specific community, in order to gain the necessary knowledge and skills and facilitate peers to make informed decisions about health related issues, and achieve a positive health outcome [17]. Existing research suggests that people are more likely to engage and change their diet-related behaviours if the educator is from their own demographic and faces similar concerns [16]. Peer educators can gather and share information in a way that is more applicable, practical and appealing to the target audience, and therefore may be more likely to result in behaviour change [18]. Previously identified advantages of using peer educators in nutrition education include cost effectiveness, culturally appropriateness and optimal use of resources [19]. The benefits of peer educator models extend to the leaders themselves, who report improvements in skill development, community status, increased caring for others and increased self-esteem [20].

Trained peer trained educators have been used effectively to influence health behaviours in a variety of settings and at various levels of intervention [21, 22]. The most widely reported use of peer facilitators has been in order to increase the uptake of health promotion messages. In a study that compared a peer developed prenatal breast feeding education program to a hospital based nurse led class, Rempel et al. [22] reported that peer facilitators had a stronger influence on mothers’ intentions to continue breast feeding their child for more than 9 months.

In a study of child feeding behaviours and attitudes of 146 parents of children aged two to five years in rural Australia, it was identified that parents believe that optimal child nutrition is vital [10], however optimal child dietary intake is difficult to attain [10]. Intention to change feeding practices was restricted by a belief that a child’s nutritional intake is above ‘average’ when compared to their peer group [10]. External factors including food advertising, peer influences and extended family perpetuated this ambivalence towards change. This reinforces the need to train mothers as peer nutrition educators in order for them to positively influence nutrition behaviours within their social groups.

In addition to helping individuals make healthy choices, information and education are essential for socialising target groups into health promoting norms and behaviours [18]. Health information can be distributed through a variety of mediums and settings. The capacity to use computer technology [23] and the Internet, through applications such as Facebook to distribute health information quickly, extensively and within specific target groups, make these potential vehicles for sharing health information [24].

We propose that embedding relevant, evidence-based child feeding and child nutrition information in a well-designed theory based peer educator model will improve the child feeding practices of parents within groups of first-time mothers. However, we need to first establish if this approach is acceptable to the target group. Therefore, this study aims to establish whether first-time mothers are interested in receiving additional child nutrition and feeding information, in what format or context they would like to receive this information, and whether peer educator delivered information would be considered an appropriate way to share and disseminate nutrition and feeding information.

## Methods

The Peer Education Nutrition Training (PeerENT) study was a cross-sectional feasibility study. Participants were recruited from an existing cohort of mothers of children aged six months to two years from the North Coast, NSW Australia who had previously subscribed to a quarterly child nutrition information email service after participating in New Parents Groups between 2010 and 2012.

The regular quarterly email was distributed to mothers by the Community Nutritionist in mid-November 2012, accompanied by an invitation to complete an anonymous online survey, which was included in the email as a hyperlink. The online survey email distribution was repeated twice within a two week period from mid to late November 2012 for those who had not responded. The survey was preceded by a participant information section, which required consent from participating mothers before accessing a brief description of the rationale for the survey and an estimation of time needed to complete the survey.

The survey consisted of 13 questions, including four demographic (maternal age, number of children, child age and postal/zip code) and nine about peer educator training as shown in Table 1.

**Table 1:** **Survey questions to determine feasibility of peer educator training in the PeerENT study**

Outcome measure	Survey question	Response options	n (%)
Current child feeding information sources	Where do you go for other information regarding feeding children? (could select more than one option)	Internet	27 (79%)
		Friend	21 (62%)
		Family	15 (44%)
		Nurse	13 (38%)
		Doctor	6 (18%)
		Dietitian	5 (15%)
		Media (magazines, newspapers)	5 (15%)
		Other (please specify)	
Current nutrition information circulation	Do you share any of the information with any of these people? (could select more than one option)	Friend	16 (47%)
		Partner	30 (87%)
		Family member	10 (30%)
		Other (please specify)	
Child feeding efficacy	Please rate your overall ability and confidence in feeding your child/ren?	Very confident	12 (35%)
		Confident	11 (32%)
		Somewhat confident	11 (32%)
		Not confident	0 (0%)
Interest in becoming a peer nutrition educator	How interested would you be in attending peer educator nutrition training?	Very interested	11 (32%)
		Interested	10 (30%)
		Somewhat interested	1 (3%)
		Not interested	12 (35%)
Time availability (total hours)	How much time are you willing to devote to peer educator nutrition training?	None	0 (0%)
		Up to 1 hour	1 (3%)
		1 – 2 hours	13 (38%)
		2 – 4 hours	5 (15%)
		4 or more hours	3 (9%)
Format of peer educator training	What format would be suitable for delivering peer educator training? (could choose more than one option)	Group	22 (65%)
		Self-directed	20 (60%)
		Online	15 (45%)
		Other	Combined
Format for peer nutrition education delivery	How receptive do you feel other parents would be about receiving nutrition information from trained peers?	Very receptive	13 (38%)
		Receptive	13 (38%)
		Unreceptive	8 (24%)
		Comments	
	What format would suit the delivery of peer nutrition education to other parents?	Structured group program	14 (41%)
		Informally in peer/ friendship group	16 (47%)
		Social media/online	17 (50%)
		Other comments	
	Please indicate the approximate number of people you are likely to share child nutrition information with?	Number _______	510 (total)

The combination of open and closed questions were developed by the research team to address the primary outcomes measures, based on the components of the Theory of Planned Behaviour. The survey was piloted for readability and participant burden with 11 mothers prior to survey implementation.

Survey Monkey (Professional version Palo Alto, California, USA), an online survey tool was used to collect and collate data that was exported for analysis to STATA statistical software (Version 10, College Station, Texas USA). Descriptive statistics including frequencies, percentages and proportions were completed, and used to produce tables and figures.

Approval for the Feeding Healthy Food to Kids study was obtained in March 2009 from Hunter New England Human Research Ethics Committee. Reference No: 08/12/17/4.02. A variation to this ethics application was approved in July 2012 to conduct a peer educator nutrition feasibility survey.

## Results

**Figure 1:**
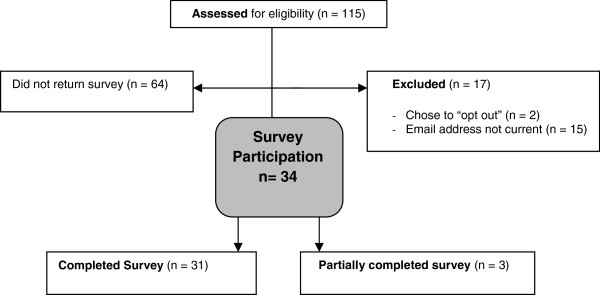
**Flow of participants through the Peer Educator Nutrition Training feasibility study.**

Surveys were emailed to 115 new mothers. Two participants chose the “opt out” option and 15 email addresses were not current, leaving 98 potential participants (Figure 1). Thirty four of the 98 eligible mothers (35%) responded to the survey, and of these 31 out of 34 participants (91%) completed the entire survey.

Fifty percent of the 34 participants were mothers aged 32–41 years (n = 17) and 41% were aged 25–31 years. Twenty seven participants (79%) had one child, six had two children and one had three children. The child age range was 6 months (11%) to over two years (47%). Demographic details are summarised in Table 2.

**Table 2:** **Demographic characteristics of participants in the Peer Educator Nutrition Training (PeerENT) study to determine the feasibility of training new mothers as peer nutrition educators**

Mother age	Responses (n = 34)
18 to 24 years	2 (6%)
25 – 31 years	14 (41%)
32 – 41 years	17(50%)
42 – 51 years	1 (3%)
Number of children	Responses (n = 34)
One	27 (79%)
Two	6 (18%)
Three	1 (3%)
Child age	Responses (n = 33)
6 - 8 months	3 (9%)
9 – 11 months	4 (12%)
12 – 15 months	0 (0%)
16 – 18 months	4 (12%)
19 – 21 months	5 (15%)
21 – 24 months	1 (3%)
Over 24 months	16 (47%)

Twenty three mothers (67%) rated themselves as confident or very confident and eleven (32%) as somewhat confident for child feeding efficacy. In descending order, participants rated a structured program (65%), social media/online (60%) and informally in peer groups (45%) as their preferred formats for the delivery of nutrition information and advice. Mothers commented that a combination of different approaches would increase the overall participation rate. *“A mix of all these would reach the greatest number of parents”, “Online is also good for some people, but I prefer talking with my mothers’ group”.*

Seventy six percent felt that other parents would be either very interested (n = 13) or interested (n = 13) in receiving nutrition information and advice on how to get their child/ren to consume healthy foods from a trained volunteer peer educator, with comments such as *“In my opinion, all tips and advice are greatly appreciated”.* One participant expressed concern about peer education, as she felt the advice given may not be based on best practice rather drawn from personal experiences, commenting that *“the parent might still let their own values in, rather than give the right advice”.*

Sixty five percent of the 34 respondents (n = 22) reported some interest in receiving additional training to learn how to share nutrition and child feeding information with other parents in an unpaid capacity, of whom 50% were very interested. Eight of the twenty two mothers were willing to devote more than 2 hours of their time to additional nutrition and child feeding training.

All mothers who expressed interest in nutrition training (n = 22) felt that it would be best if the nutrition and child feeding training was in a structured group session with other parents, with seven parents also indicating that online training would be acceptable. However, after receiving the training, the preferred methods of communicating this information to other parents were; online (n = 17), informally in a peer or friendship group (n = 16) and in a group program (n = 14). The preference for online training was supported by comments such as “*Working full time with 2 children, online info is great!”*

The new mothers in this study cited the Internet (n = 27), friends (n = 21) and family (n = 15) as their usual sources of nutrition information. Health professionals were less often cited sources of nutrition information, with nurses cited by 13 participants, doctors by 6 and dietitians cited by 5 participants. Eighty seven percent of mothers shared nutrition information that had been provided to them with their partners and 47% with their friends. The mean estimated number of contacts which each participant indicated they would share information with was fifteen, or a total reach of 510 from this study cohort, excluding potential overlap between participant social networks.

## Discussion

Despite the time pressures and stresses associated with parenthood [25, 26], participants seem to be motivated to seek nutrition information for their own purposes. Responses to this survey demonstrated an encouraging level of interest by mothers in participating in a peer educator nutrition training (Peer ENT) program, both as participants and as peer leaders. They were equally willing to devote their personal time to undertake nutrition training in peer leadership and to share this information within their parenting peer groups.

Results of this study reinforce the results of previous research into child nutrition education for new parents [27] and identified the potential for further research into peer educator training for nutrition education. The willingness of mothers in this study to become peer educators was consistent with the success of “train-the-trainer” models in other study populations [19–21]. An unexpected positive finding was the willingness of mothers with young children to devote two hours or more of their time to participate in peer educator training. New mothers are notoriously time-poor and difficult to reach as a target group [26], so this result provides evidence of these parents’ motivation and commitment to child feeding and nutrition. This result is consistent with previous research indicating that changes in stage of life constitute opportunities to engage people in behaviour change [28].

While the majority of parents were supportive of peer educator training, some resistance was expressed by participants who felt that peer education may impact the quality and consistency of information being provided. This concern reflects those expressed in previous studies in which peer educator models have been developed, implemented and evaluated [19–21, 24, 27]. It is therefore imperative that the nutrition education content of peer educator training is appropriate for delivery by lay population, and incorporate evidence-based peer education principles.

Use of Internet, both as the participant’s current source of nutrition information and their desired mode of delivery of peer education, inform the direction of future research. The challenge of ensuring the integrity of nutrition information supplied and received via the Internet can be addressed by providing peer educators with simple, informative, succinct child nutrition resources that can easily be shared via the Internet [24]. The viability of the peer education process may be further enhanced by ensuring that an Accredited Practicing Dietitian is available as a support resource for peer educators, and this needs further research.

Dissemination of information to peers in online forums provides a potentially effective combination of peer education delivery to friends, with the preference for Internet or social media as a medium. This finding suggests that a model for further research would combine face-to-face peer nutrition educator training, to train as many “peers” in new parent cohorts as possible, with quality resources that can be shared via social media and other electronic mediums. The predicted capacity of participants to share the nutrition resources and information with an average of fifteen people, shows that the reach of peer nutrition education is at least ten times the capacity of individual interactions.

Limitations to the study include the use of self-reported data, potentially resulting in social desirability reporting bias. The low response rate was nevertheless consistent with other studies using online surveys [29] and studies that included surveys of new mothers [30]. The study cohort was small, predominantly mothers and recruited from a regional area, which could influence the generalisability of the results into other population groups. The potential for using peer educator training for new fathers, and less motivated parents requires further investigation. However, given that this study was aimed at establishing the feasibility of peer educator training, the results were of adequate strength to support a pilot peer educator nutrition training program.

## Conclusions

The results of the current survey indicate support for the development and testing of a child feeding and nutrition program to determine the effects of a well designed, theory based peer educator model on the child feeding practices of parents. The peer educator training needs to be relevant to parents with children aged between six months to three years and have face-to-face and self-directed delivery options.

It is proposed that in areas where new parent groups exist, this forum provides an ideal avenue for offering and implementing peer nutrition education. In areas where new parent groups are not available or accessible, it is possible that peer educator nutrition training could be arranged in collaboration with early childhood health service providers or community based agencies and interest groups such as breastfeeding support groups, or provided in an online format.

The finding that new mothers would prefer to deliver nutrition messages to their peers via social media is important, with obvious implications for the type of resources that need to be developed for the peer educators. Researchers who are planning to conduct peer educator training need to collaborate with information technology experts to develop resources that can be disseminated appropriately through social media and other online formats. Nutrition resources that are disseminated using the Internet via peer educators needs to be identifiable as being from a reputable source.

Considerations of online technologies, such as online blogs or the use of avatars are potential applications. This could appeal to the first-time parents, who are entering a new phase of life and may feel isolated. The use of interactive computer technology by trained peer educators in conjunction with face-to-face education sessions and on-line sharing of nutrition information within and amongst peer groups is a promising avenue of future research.
